# Supplementary material for: New seasonal measurement with stability and clustering seasonal patterns: A case study in Japan from 2011 to 2019
Source: PLoS One. 2022 Apr 22;17(4):e0267453. doi: 10.1371/journal.pone.0267453 (PMC9032376; doi:10.1371/journal.pone.0267453)
Supplement: Attachment — Submitted filename: Response to Reviewers.docx [file pone.0267453.s001.docx]

Response to Reviewer #1:

I wish to express my appreciation to the reviewer for insightful comments on our manuscript. The comments have helped significantly improve the paper.

The following is a point-by-point response to the reviewers' comments.

1. The literature of this article is poor. It is recommended to provide the literature on the topic. The motivation behind the topic also needed to include.

Thank you for your thoughtful suggestion. I added some references on this topic to express more details and clarify the motivation. Specifically, I added sentences on lines 76-85, 144-147, 151-159 and 170.

2. It is recommended to summarize the contributions in the introduction.

I agree with your suggestion. I added a paragraph to mention the contribution of this study on lines 86-94. This contribution includes a result of the comparison study which is newly added in the revised manuscript.

3. Section 3 is too small, it is recommended to elaborate through detailed discussion and importance of each equation.

Thank you for your suggestion. I explained Eq. (3) on lines 182-184 and added sentences and a figure on lines 192-201 to discuss for details.

4. There are several performance metrics in the literature, whereas the authors considered only few. It is recommended to evaluate through multiple metrics.

Since the individual data (prefectures) in the Japanese case study does not have correct answer labels, it is difficult to derive performance metrics for an evaluation. Hence, I additionally conducted a clustering analysis with traditional seasonal index and Ward’s method to compare with the results of a suggested method and evaluate by obtained insights. I explained these results in the section named “Using traditional seasonal indices” in page 16 and on lines 411-416 and 426-439.

5. It is recommended to provide the futuristic prediction about the seasons.

I appreciate your comment on this point. Accordingly, I have added sentences on lines 390-392 and lines 423-425. I have mentioned the futuristic prediction of Japanese seasonality, based on the results of this study.

6. What are the limitations identified during your study?

I thank the reviewer for this comment. I additionally mentioned the limitations on lines 466-468 and 474-478.

Response to Reviewer #2:

I wish to thank the reviewer for thoughtful comments on my manuscript. The comments have helped significantly improve the paper. The following is a point-by-point response to the reviewers' comments.

1. Need to add a Comparison study.

I wish to thank for this comment. The results of the additional work are shown in the section named “Using traditional seasonal indices” with Fig 15, 16, 17 and 18 in page 16. The discussion for the results were mentioned on lines 411-416 and 426-439.

2. Need to update the limitation.

Thank you for this comment. I additionally mentioned the limitations on lines 466-468 and 474-478.

3. How to deploy the research idea, detailed information required.

I appreciate the comment on this point. I added a future issue and its details on lines 479- 487.

4. In many figures the X-Axis name was inverted, kindly reupdate it.

Thank you for the review’s suggestion. I confirmed that many figures were automatically rotated when making a combined pdf file in the submitting process. Specifically, Fig. 1, 2, 3, 4, 5, 6, 7, 8, 9, 10, 11, 12, 17 and 18 were rotated at 90 degrees in the resubmitted pdf file. I consider that this problem might be caused by the submitting system.

5. Overall the idea was good.

6. The dataset is available in public, easily we can able to downloadable. That's great work.

Thank you so much for these comments. As the review’s pointing out, all results in this study are derived from Japanese government open data. I am glad to hear that.
